# Telemedicine and Gestational Diabetes Mellitus: Systematic Review and Meta-Analysis

**DOI:** 10.7759/cureus.71907

**Published:** 2024-10-20

**Authors:** Omnia S El Seifi, Faten E Younis, Yassin Ibrahim, Shamina B Begum, Salwa F Ahmed, Eman S Zayed, Iman Mirza

**Affiliations:** 1 Family and Community Medicine, University of Tabuk, Tabuk, SAU; 2 Microbiology, University of Tabuk, Tabuk, SAU; 3 Anatomy, Faculty of Medicine, University of Tabuk, Tabuk, SAU; 4 Clinical Biochemistry, Faculty of Medicine, University of Tabuk, Tabuk, SAU

**Keywords:** fetal outcomes, gestational diabetes mellitus, maternal outcomes, randomized controlled trials, telemedicine

## Abstract

Hyperglycemia known as gestational diabetes mellitus (GDM) can happen during pregnancy and poses a risk to the developing baby as well as the mother. Glycemic control, patient involvement, and diabetes management might all be improved via telemedicine (TM). Therefore, this study aimed to compare TM versus standard care for GDM in terms of maternal and fetal outcomes.

The authors searched for randomized controlled trials (RCTs) contrasting TM with conventional care among gestational diabetes women across various databases including PubMed, the Cochrane Central Register of Controlled Studies, and Google Scholar from April 2010 to December 2023. This meta-analysis included a total of 2,192 pregnant women from 12 RCT studies and was analyzed by RevMan (version 5.4; Cochrane, London). Applying fixed and random effects was based on heterogeneity.

There was a statistically significant difference in the effect on the control of blood glucose levels two-hour postprandial (MD = −0.45, 95%CI = (−0.84, −0.06), P = 0.02) and on the cesarean section effect (RR = 0.74, 95%CI = (0.63, 0.87), P < 0.001) when TM was compared to standard care for GDM. However, there was no statistically significant difference in the effect on other maternal or fetal outcomes such as HBA1c, fasting blood glucose, preterm birth, fetal macrosomia, or hypoglycemia.

TM interventions are more successful than standard therapy in lowering the rate of cesarean section and decreasing the two-hour postprandial glucose level of GDM patients, which is essential for improving glycemic control and reducing cardiovascular disease.

## Introduction and background

The term “gestational diabetes mellitus” (GDM) refers to any degree of glucose intolerance that appears or is diagnosed during pregnancy [1]. Globally, GDM affects 14% of expectant mothers; however, numbers vary due to variations in screening methods and diagnostic standards [2]. Most pregnant women with GDM do not have any symptoms, while vomiting, nausea, frequent urination, bladder infections, and fungal infections are possible adverse effects [3].

Uncontrolled conditions of DM during pregnancy can be harmful to the developing fetus as well as the mother. Cesarean delivery, polyhydramnios, abortion, pre-eclampsia, early labor, placenta previa, infection of the urinary tract, and puerperal sepsis are examples of maternal side effects [4,5]. Maternal GDM is linked to macrosomia and the pathophysiological effects of fetal hyperglycemia and hyperinsulinism, which puts the fetus and infant at risk [6]. Type 2 diabetes mellitus (T2DM) is more common in females with GDM relative to the overall population. The incidence of T2DM and chronic illnesses in children born to mothers with the condition may be decreased by stopping the course of GDM [3].

Pregnant women with GDM should promptly report their blood glucose dynamics to healthcare specialists. The standard care for patients with GDM involves expecting mothers to check their blood sugar levels and manually recording them in paper logbooks multiple times a day at their homes. Following this, medical professionals assess the glucose data and offer health education during routine antenatal exams. The conventional approach to standard care has certain drawbacks, including incomplete patient and physician connections and delayed information [7]. The field of information and communication technology has recently developed at a rapid pace, offering new managerial and technical support options to enhance care and pregnancy outcomes for mothers with GDM [8,9].

The term “Telemedicine” (TM) describes medical and health services processes, including remote patient assessment, diagnosis, and treatment by medical experts. These procedures are carried out using distant communication technologies, which include websites, email, Bluetooth, mobile phones, and phones [10,11]. According to the technology used, the TM has different forms, including mobile health (mHealth), videoconferences, game-based support, social platforms, and patient portals [9,12,13].

TM has remarkable benefits for the healthcare system, especially related to GDM; Healthcare providers use TM tools to keep an eye on patients' health-related indicators, provide prompt medical feedback, and remotely impart health knowledge and advice to enhance patients' mental and physical well-being [10,11].

From the patient side, TM helps with the clinical management of mothers with GDM, especially those in remote places, to easily access medical consultations, health education, remote monitoring, digital health coaching, enhancing glycemic control and patient engagement, and real-time uploads of glucose data, symptoms, and indicators [14]. For the health care system, TM ensures efficient use of medical resources, decreases waiting time, and improves the quality of health care [12].

Despite the many benefits of TM, especially in the context of the COVID-19 pandemic, certain barriers still hinder its widespread use in healthcare disease management. These include a lack of clear guidelines for its application, inadequate training for patients and healthcare providers, incorrect diagnosis, and a low level of physician acceptance of its use in disease management [15,16]. There are some discrepancies in the results of studies exploring the effect of TM on GDM; many of them found that using TM tools was more successful than standard therapy in improving mother and fetal outcomes [8,10], while another study concluded that TM has no significant effect on reducing pregnancy complications [17].

Reviewing literature from different primary studies in a systematic review and statistically condensing contradictory results in the meta-analysis is important to assess the efficacy of intervention techniques that enhance patient outcomes. As a result of the growing widespread use of digital technology in the field of medicine and as a trial to resolve the variations in the evidence about the effect of using TM in the management of GDM cases, this study was conducted to compare TM versus standard care for GDM in terms of maternal and fetal outcomes.

## Review

Materials and methods

To conduct this study, the authors adhered to the Preferred Reporting Items for Systematic Reviews and Meta-Analysis (PRISMA) standards [18]. The selection of research was based on PICOT, which consisted of patient population, intervention, comparator, outcome, and timing. The authors searched the database for randomized controlled trials (RCTs) comparing pregnant women with gestational diabetes receiving TM intervention versus those with standard care from April 1, 2010 to December 31, 2023, and used PubMed, Cochrane Central Register of Controlled Trials (CENTRAL), and Google Scholar. Only articles that have been published in English were considered. A combination of TM-related terms (Virtual Medicine, Medicine, Virtual, Telehealth, eHealth, Telecare, Tele-Care, TeleCare, Mobile Health, Health, Mobile, mHealth, web-based, smartphone, smartphone, cell phone, internet) and terms related to GDM (gestational hyperglycemia, hyperglycemia in pregnancy, pregnant diabetes) made up the search terms using Boolean operators; AND, OR, and NOT.

Selection criteria

Inclusion Criteria

Only studies published in English were included, free whole-text research, and RCT study design where pregnant GDM patients in the experimental group received TM therapies and those in the comparison group received standard care were among the types of studies that were searched.

Exclusion Criteria

The duplicate publications, enrollment of women with diabetes type I or type II, non-RCTs, studies with subdivisions for the TM group, and studies with limited data extraction were the criteria used to exclude research papers from the analysis.

Quality Assessment

Three authors (O.S.E., F.E.Y., Y.I.) separately sifted through the literature, gathered information, and assessed the included study for possible bias. If there was a disagreement, it was resolved through discussion. Each article's title and abstract were revised to eliminate any that were obviously inappropriate. Subsequently, the authors scanned the entire document to pinpoint the relevant research. Most of the information that was extracted was divided into three categories: (a) the core components of the literature, such as the sample size, country or region, year of publication, first author, etc., (b) the maternal outcomes, and (c) the fetal outcomes.

Risk of Bias

Two authors (O.S.E. and F.E.Y.) assessed the risk of bias in the selected articles using version 2 of the Cochrane risk-of-bias instrument for randomized trials (RoB 2) [19].

Statistical Analysis

RevMan (version 5.4; Cochrane, London) was utilized to analyze the continuous and categorical dichotomous data to compare pregnant women with GDM who obtained TM intervention versus those who received standard care. Heterogeneity was the basis for the application of fixed and random effects. I^2^ values >50% are considered to indicate substantial heterogeneity (reference 10). P-values less than 0.05 were deemed significant.

Results

**Figure 1:**
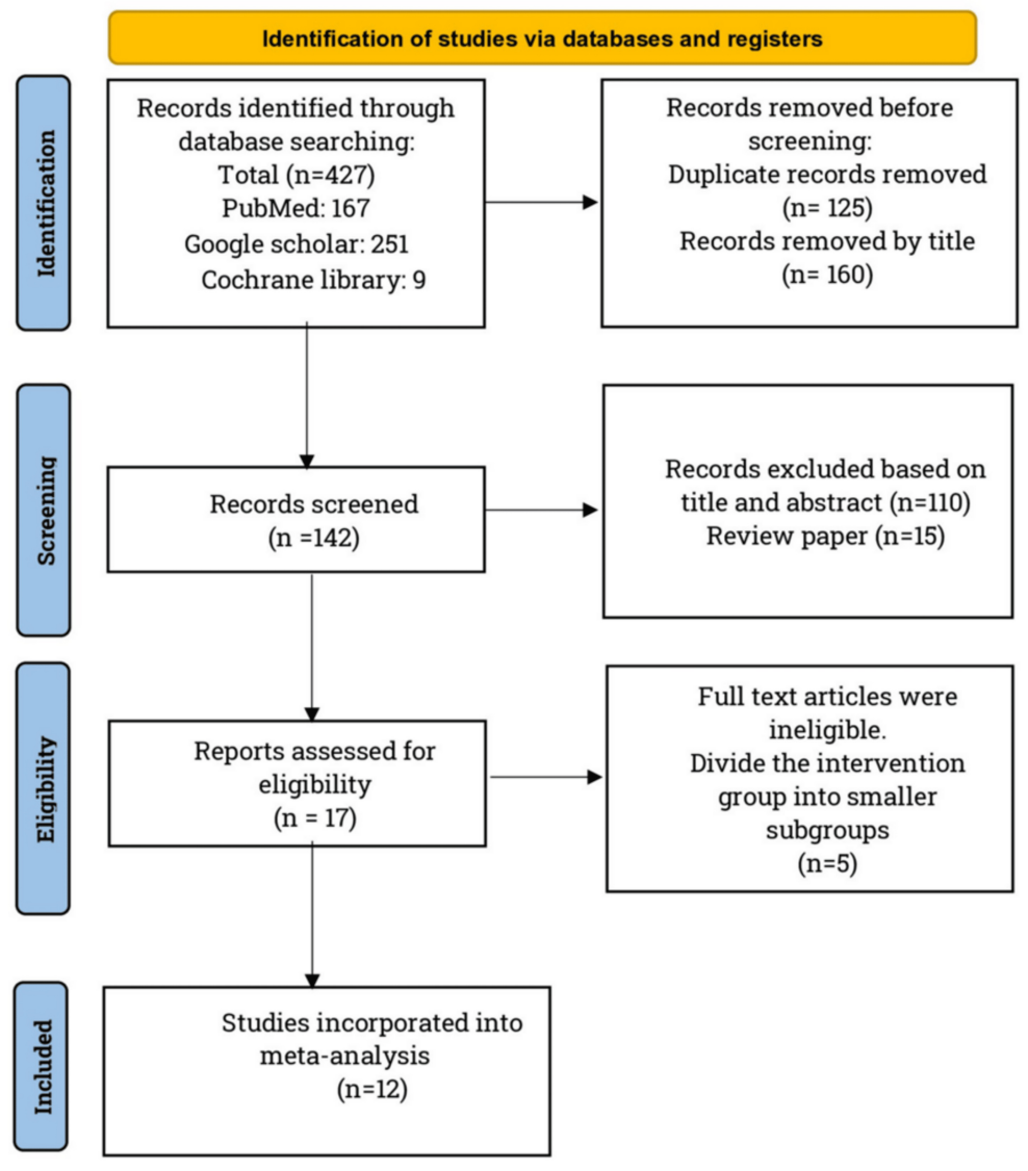
PRISMA flow diagram. PRISMA: Preferred Reporting Items for Systematic Reviews and Meta-Analyses

By applying the inclusion and exclusion criteria, 427 RCT publications were found in three databases. Of these, 12 studies were chosen for meta-analysis (Figure 1). A total of 2,192 pregnant women were included in this study: 1,097 in the TM group and 1095 in the standard care group. Most of the included studies were conducted in China (48.1%). The minimum sample size was 80 and the maximum was 410 participants. The details of the selected research articles are presented in Table 1.

**Table 1:** Characteristics of the studied research. HbA1c: glycated hemoglobin; FBG: Fasting blood glucose; CS: Cesarean section

Studied research	Region	Total participants	Telemedicine participants	Standard care participants	Intervention	Extracted outcomes
Guo et al., 2019 [8]	China	124	64	60	The Mobile health (mHealth) application on smartphones.	HbA1c, FBG, two-hour postprandial, CS, macrosomia and fetal hypoglycemia
Munda et al., 2023 [20]	Slovenia	105	53	52	A smartphone application, that simultaneously transfer capillary glucose levels.	HbA1c, FBG, two-hour postprandial, CS, preterm birth, macrosomia and fetal hypoglycemia
Pérez-Ferre et al., 2010 [21]	Madrid, Spain	97	49	48	A central database and outlying devices, such as cell phones and a Glucometer with infrared capability that can transmit data.	HbA1c, preterm birth
Chen et al., 2021 [22]	China	139	79	60	The exercise-based, personalized nursing care in conjunction with the internet.	FBG, two-hour postprandial, CS, and preterm birth
Homko et al., 2012 [23]	USA (United State of America)	80	40	40	A phone system with integrated interactive voice response (IVR) and web-based informatics applications.	Fasting blood glucose, two-hour postprandial glucose, CS, preterm birth
Tian et al., 2021 [24]	China	269	133	136	WeChat group management.	FBG, two-hour postprandial, CS, preterm birth, and macrosomia
Khorshidi Roozbahani et al., 2015 [25]	Iran	80	40	40	Telephone intervention.	Fasting blood glucose
Shao et al., 2018 [26]	Shanghai, China	410	205	205	WeChat intervention.	Two-hour postprandial glucose
Borgen et al., 2019 [27]	Oslo, Norway	233	112	121	An application on mobile phones.	CS, macrosomia
Chen et al., 2022 [28]	China	112	51	61	Interactive continuous nursing intervention on the WeChat platform.	CS, preterm birth and macrosomia
Mackillop et al., 2018 [29]	UK (United Kingdom)	203	101	102	An application on a mobile device (GDM-health) for glucose level.	CS, preterm birth, fetal hypoglycemia
Yew et al., 2021 [30]	Singapore	340	170	170	The lifestyle coaching program runs on the Web/Smartphone.	CS, preterm birth, macrosomia and fetal hypoglycemia

HbA1c Control

**Figure 2:**
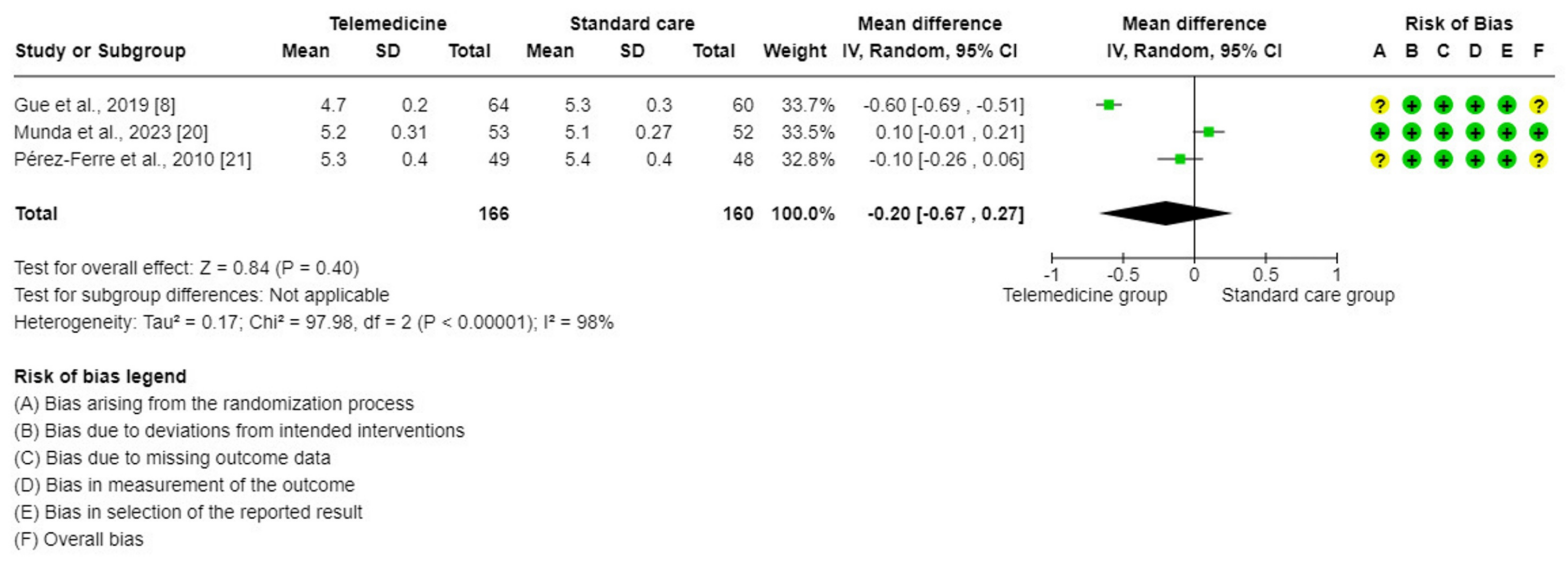
Forest plot for comparing HbA1c control between telemedicine and standard care groups.

The forest plot of meta-analysis of three trials [8,20,21] showed that there is no significant difference in the effect on HbA1c levels when comparing TM to standard care for GDM (mean difference (MD) = −0.20, 95%CI = (−0.67, 0.27), P = 0.40). There was a high degree of significant variability between the studies included in this analysis (I2 = 98%, P < 0.001). Therefore, a random effect method was used for analysis (Figure 2). The sensitivity analysis illustrates that there was only a slight change in the pooled effect and I^2^ statistic following the item-by-item exclusion.

Fasting Blood Glucose Control

**Figure 3:**
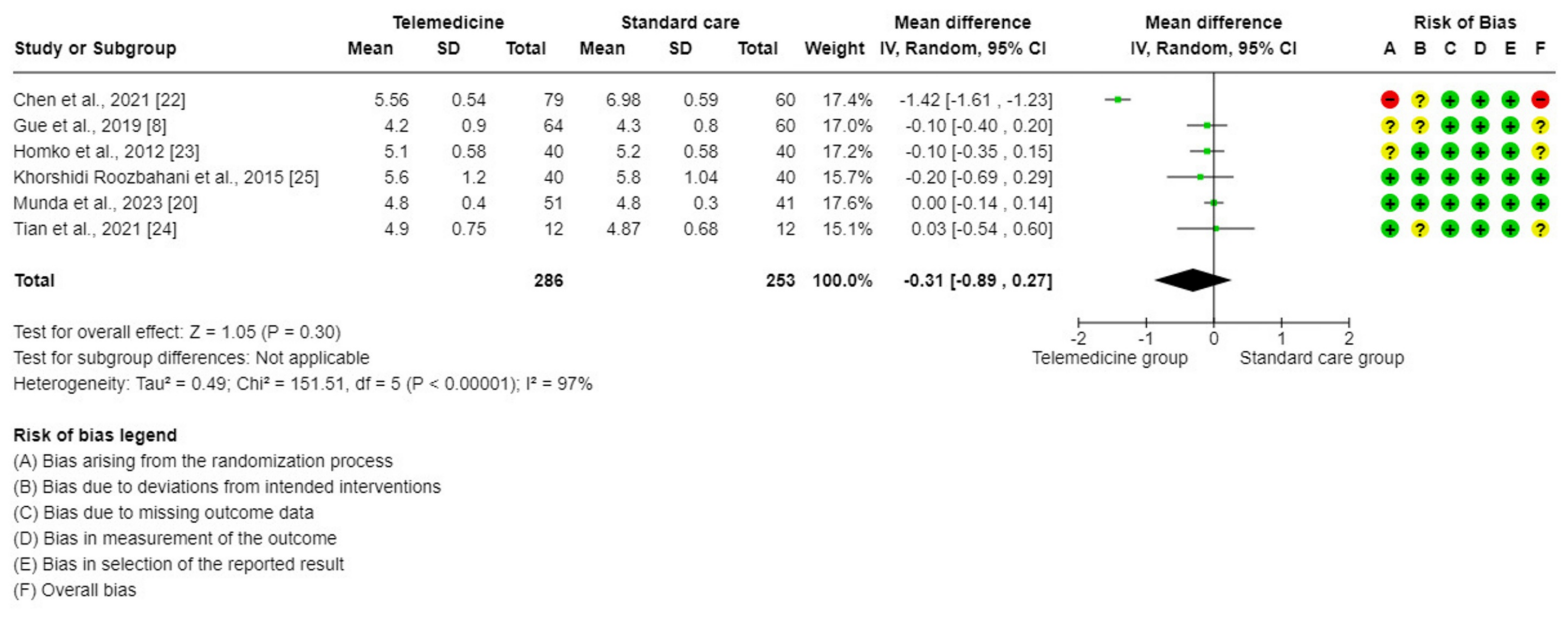
Forest plot for comparing fasting blood glucose control between telemedicine and standard care groups.

The meta-analysis of six trials [8,20,22-25] showed in the forest plot that the difference in the effect on fasting blood glucose levels when comparing TM to standard care for GDM was not significant (MD = −0.31, 95%CI = (−0.89, 0.27), P = 0.30). There was a high degree of significant variability between the studies included in this analysis (I2 = 97%, P < 0.001). Therefore, a random effect method was used for analysis (Figure 3). The sensitivity analysis revealed that the Chen et al. [22] trial was the primary cause of the heterogeneity and that after it was eliminated, the heterogeneity decreased to 0.0%.

Two-Hour Postprandial Glucose

**Figure 4:**
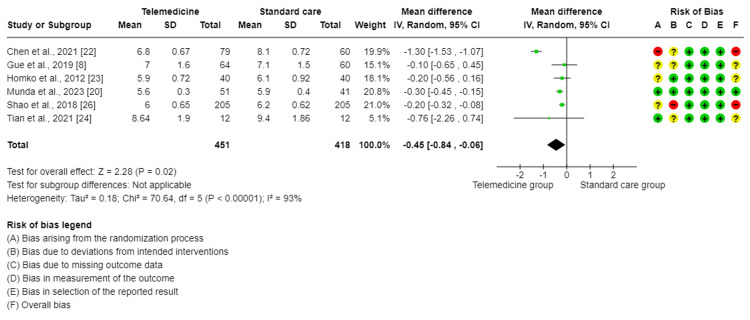
Forest plot for comparing two-hour postprandial glucose control between telemedicine and standard care groups.

The meta-analysis of six trials [8,20,22-24,26] suggested through this forest plot that there was a statistically significant difference in the effect on the two-hour postprandial blood glucose levels when comparing TM to standard care for GDM. TM groups showed a better effect (MD = −0.45, 95%CI = (−0.84, −0.06), P = 0.02). There was a high degree of significant variability between the studies included in this analysis (I^2^ = 93%, P < 0.001). Therefore, a random effect method was used for analysis (Figure 4). The sensitivity analysis demonstrates that the Chen et al. [22] trial was the primary cause of the heterogeneity and that after it was excluded, the heterogeneity dropped to 0.0%.

Maternal and Fetal Outcomes

**Figure 5:**
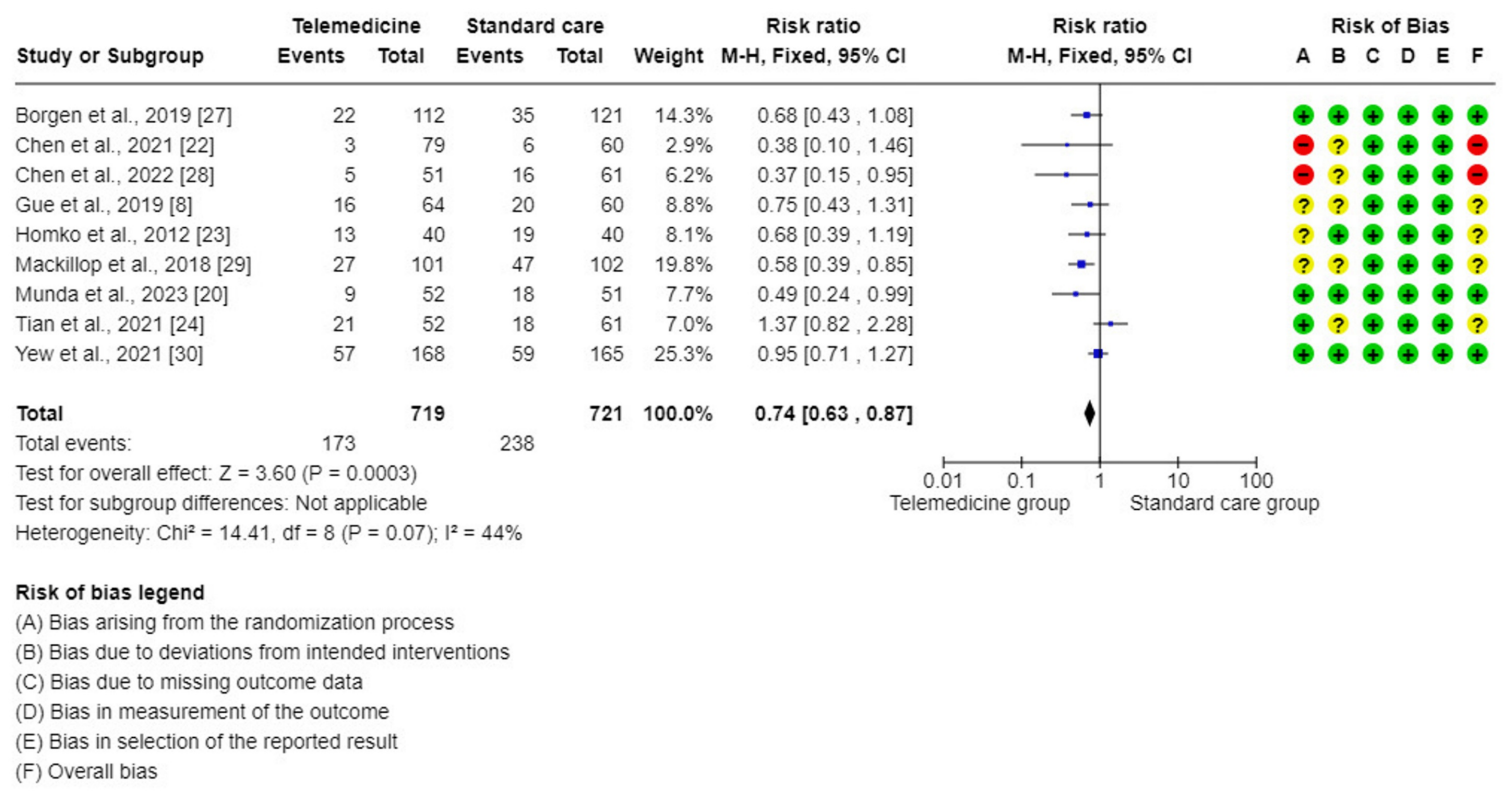
Forest plot for comparing cesarean section (CS) between telemedicine and standard care groups.

The meta-analysis of nine trials [8,20,22-24,27-30] showed that this forest plot had a significant difference in the effect of cesarean section when comparing TM to standard care for GDM. TM groups showed better effect (Risk Ratio (RR) = 0.74, 95%CI = (0.63, 0.87), P < 0.001). There was a low degree of insignificant variability between the studies included in this analysis (I2 = 44%, P < 0.07). Therefore, a mixed method was used for analysis (Figure 5).

**Figure 6:**
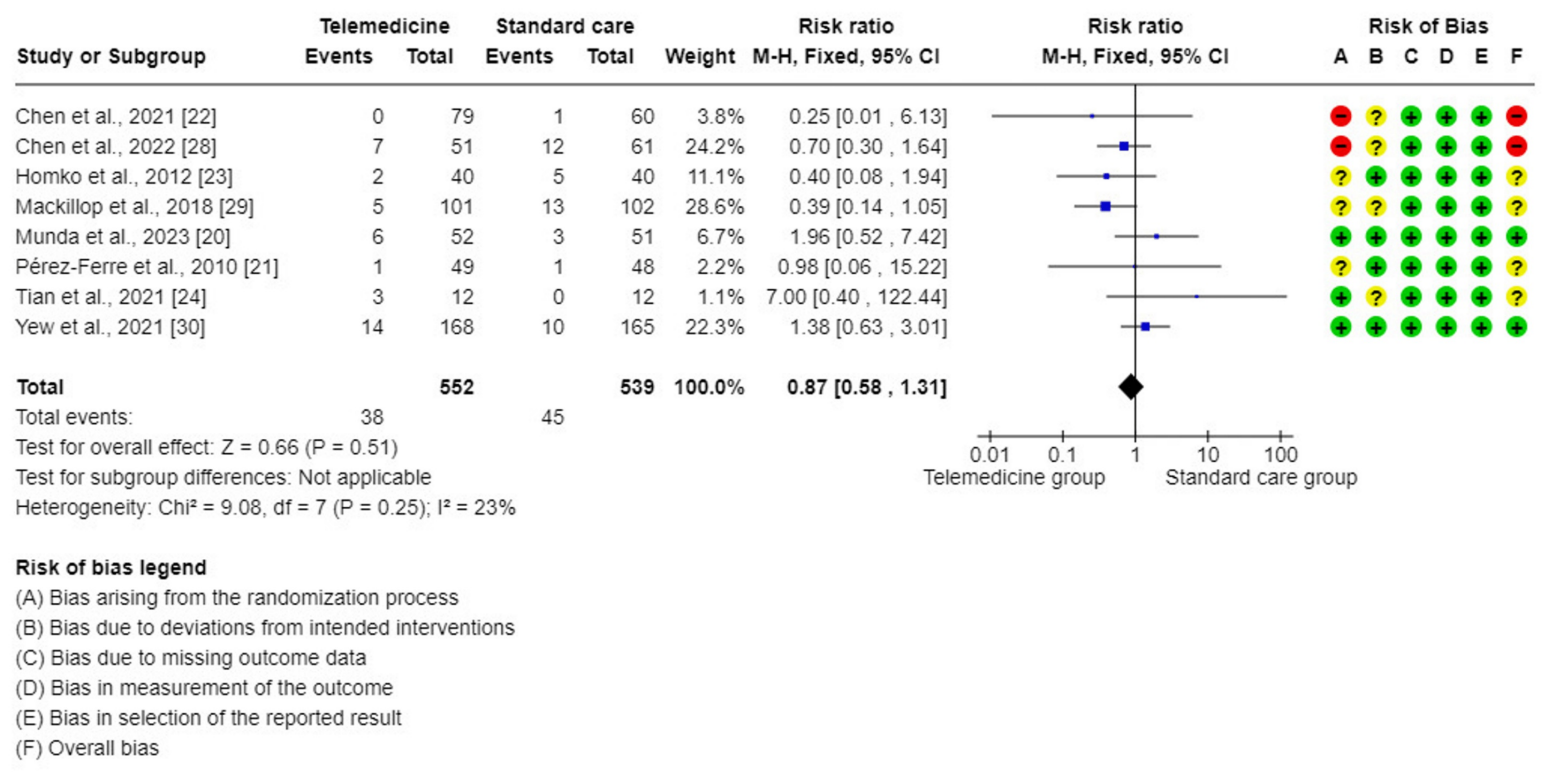
Forest plot for comparing pre-term birth between telemedicine and standard care groups.

The meta-analysis of eight trials [20-24,28-30] reported that this forest plot had no significant difference in the effect on pre-term birth when comparing TM to standard care for GDM (RR = 0.87, 95%CI = (0.58, 1.31), P = 0.51). There was a low degree of insignificant variability between the studies included in this analysis (I^2^ = 23%, P < 0.25). Therefore, a mixed method was used for analysis (Figure 6).

**Figure 7:**
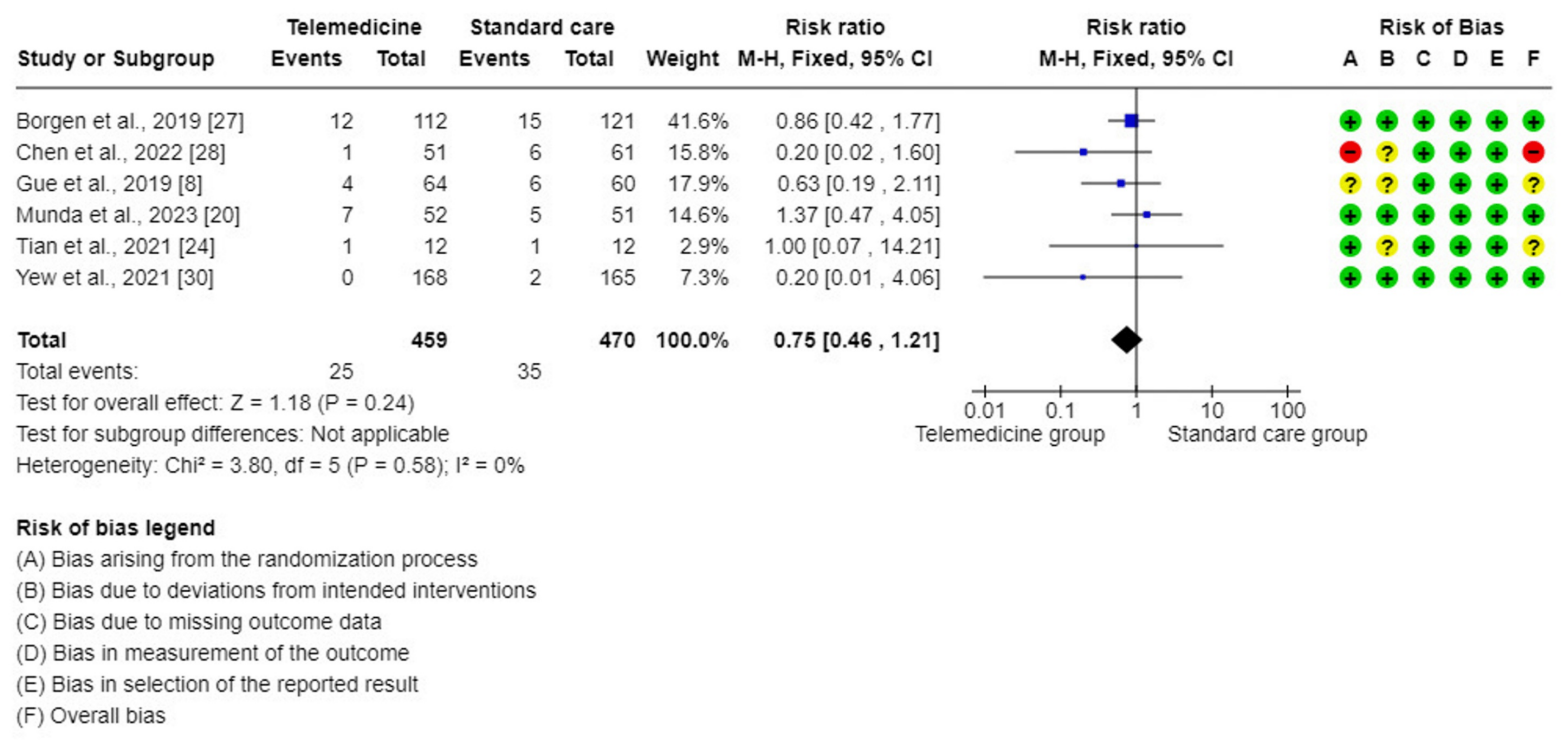
Forest plot for comparing macrosomia between telemedicine and standard care groups.

The meta-analysis of six trials [8,20,24,27,28,30] showed that this forest plot had no significant difference in the effect on macrosomia when comparing TM to standard care for GDM (RR = 0.75, 95% CI = (0.46, 1.21), P = 0.24). There was no significant variability between the studies included in this analysis (I^2^ = 0.0%, P = 0.58). Therefore, a mixed-effect method was used for analysis (Figure 7).

**Figure 8:**
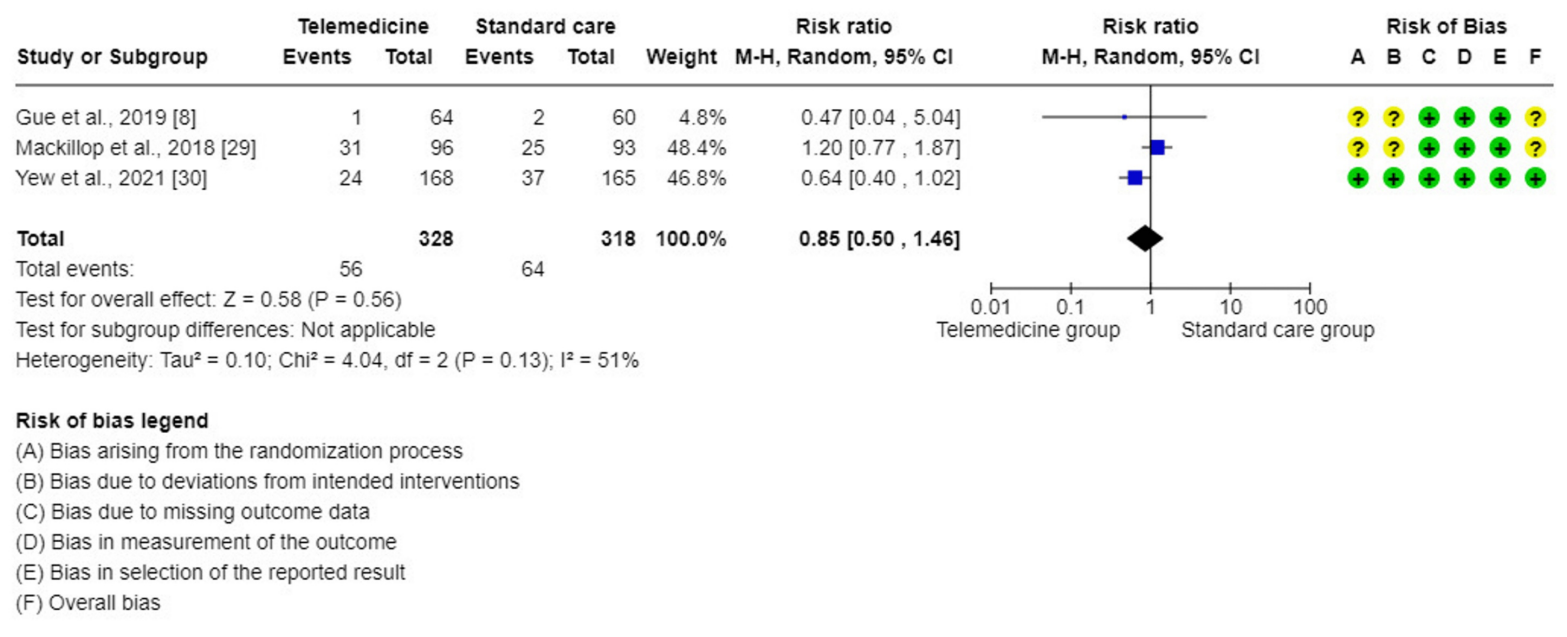
Forest plot for comparing fetal hypoglycemia between telemedicine and standard care groups.

The meta-analysis of three trials [8,29,30] showed that this forest plot had no significant difference in the effect on fetal hypoglycemia when comparing TM to standard care for GDM (RR = 0.85, 95% CI = (0.50, 1.46), P = 0.56). There was a moderate degree of variability between the studies included in this analysis (I^2^ = 51%, P = 0.13). Therefore, a random effect method was used for analysis (Figure 8). According to the sensitivity analysis, the heterogeneity was primarily caused by Mackillop et al.'s study [29] and reduced to 0.0% when this study was eliminated.

Discussion

GDM raises the risk of preterm birth, fetal macrosomia, fetal hypoglycemia, cesarean section, preeclampsia, and other pregnancy-related problems for both the mother and the fetus. Thus, conducting effective control of maternal blood sugar during pregnancy is extremely important for the health of pregnant mothers and babies [7].

In comparison with the routine mode of health delivery, TM interventions in their different forms are more adjustable and convenient in providing contents about GDM, physical activity, and healthy diet to pregnant women with less consumption of time and effort for the pregnant ladies and easy and rapid communication with the health care providers [31].

Regarding the effect of TM on the glycemic control of pregnant women with GDM, this study’s results illustrated only statistically significant lower two-hour postprandial blood glucose levels within TM care when compared to standard care (MD = −0.45, 95%CI = (−0.84, −0.06), P = 0.02), while no significant difference was present between the two groups regarding HB A1c and fasting blood glucose ((MD) = −0.20, 95%CI = (−0.67, 0.27), P = 0.40) and (MD = −0.31, 95%CI = (−0.89, 0.27), P = 0.30), respectively. This is typically the same as the findings from a study conducted by Al-ofi et al., who found that two-hour postprandial glucose was lowered significantly in the TM group than the usual care group (p = 0.002) while fasting glucose and HBA1c were not significantly affected by different types of care [32].

According to Rossetti et al. [33] and Ceriello [34], postprandial glucose levels have a significant role in glycemic control and can direct diabetic treatment. Additionally, it has been determined that postprandial glucose represents an independent risk factor for heart disease [35].

The results from this meta-analysis demonstrated that the use of TM interventions diminished significantly the frequency of cesarian section (CS) (RR = 0.74, 95%CI = (0.63, 0.87), P < 0.001). This is in agreement with the result of other similar meta-analyses that demonstrated the positive effect of TM in lowering CS among pregnant women with GDM such as Xie et al. (RR = 0.82, P = 0.02) [14], Wei et al. (RR = 0.67, 95% CI = (0.52, 0.88), P = 0.004) [36], and Leblalta et al. (RR = 0.81, CI = 0.69 to 0.95, P = 0.009) [37].

Regarding other maternal and fetal outcomes such as pre-term birth, macrosomia, and fetal hypoglycemia, they did not differ significantly in the TM group compared to the standard care group in this study. This is in line with other studies conducted by Ming et al. [10], Miremberg et al. [17], and Leblalta et al. [37].

The results of this study are different from those of other similar studies, as many of them found that using TM tools was more successful than the standard tools in lowering glycemic indices; Guo et al. and Ming et al. revealed the significant effect of TM on reducing glycated hemoglobin (HbA1c) only (8, 10). While other studies demonstrate the success of TM in lowering all maternal glycemic indices [14,36-38].

For the maternal and fetal outcomes, all were significantly lowered by the effect of TM intervention in two previous studies [14,36]. However, a meta-analysis conducted by Rasekaba et al. [38] disclosed that TM has no significant effect on glycated hemoglobin (HbA1c) nor two-hour postprandial glucose of the mother, as well as no effect on maternal or fetal outcomes compared to pregnant mothers receiving standard care.

The discrepancy in the results between these meta-analysis findings and the others could be attributed to the difference in the number of search databases and search strategies and inclusion and exclusion criteria for the selection of manuscripts. Nevertheless, we can state that there is an opportunity for the application of TM technology in managing women with gestational diabetes.

Implications of This Study for the Clinical Practice

TM technology helps monitor glucose levels, reducing the potential for invasive methods used several times a day. This will enhance patients' quality of life and offer standard information in a clinical setting for evaluation. It would also be a desirable approach to optimizing healthcare resources. Among women with GDM, TM technology has emerged as a useful tool and a potential future development trend. Using this technology, patients with GDM can benefit from long-term blood glucose monitoring and data sharing, all of which have positive societal effects. Consequently, regular collaboration between medical professionals and patients, in addition to technological advancements, must be further improved to encourage involvement.

Strengths and limitations of the study

This study only includes RCT trials, which are higher in the evidence hierarchy. In addition, covering diverse countries and regions around the world provides a more global perspective while examining the health problem. Furthermore, the results of this research revealed that TM has a beneficial influence on postprandial glucose regulation, which is essential for improving glycemic control and reducing cardiovascular disease.

The limitations of this analysis include trials with diverse approaches to TM, different sample sizes, variable durations, intervention content, and frequency, and different nations with varying availability to TM technology, which may result in heterogeneity and inconsistency of the results.

## Conclusions

When the TM approach among pregnant women with gestational diabetes was compared to standard care, two-hour postprandial glucose and the rate of cesarean delivery were found to be significantly lower among the TM group. This highlighted the promising effect of TM use on GDM and the possibility of utilizing such cost-effective technology to improve patients' quality of life. Subsequently, further studies are required to investigate the long-term outcomes of TM in GDM management or the impact of different TM modalities.
